# Molecular characterization of the TUBG1 meshwork's influence on Cytoskeletal organization

**DOI:** 10.1016/j.heliyon.2025.e41829

**Published:** 2025-01-15

**Authors:** Darina Malycheva, Maria Alvarado-Kristensson

**Affiliations:** Molecular Pathology, Department of Translational Medicine, Lund University, SE, 21428 Malmö, Sweden

## Abstract

The γ-tubulin (TUBG) meshwork is a central regulator of cellular architecture, orchestrating processes such as microtubule nucleation, mitochondrial organization, and genomic integrity. This study investigates the molecular impact of TUBG depletion on the cytoskeleton. Knockdown of TUBG using single guide RNA disrupted microtubule, vimentin, and lamin B networks while simultaneously reinforcing actin filaments structures. These findings suggest that actin reinforcement may act as a compensatory response to the broader disruption of cytoskeletal integrity. Expression of N-terminal (TUBG^1-335^) or C-terminal (TUBG^334-451^) fragments of TUBG1 partially restored these networks, with the C-terminal fragment demonstrating greater effectiveness reestablishing microtubule integrity. Both fragments stabilized vimentin filaments and the nuclear envelope, underscoring TUBG's dual structural and regulatory roles across multiple cytoskeletal systems. This study highlights the critical hubbing properties of TUBG in coordinating cytoskeletal integrity and its potential as a therapeutic target in cytoskeleton-related disorders.

## 1 Introduction

The organization of cellular components into distinct compartments, such as the nucleus, mitochondria, and cytoskeleton, is crucial for coordinating cellular activities, maintaining homeostasis, and regulating biochemical processes [1]. The tubulin family of proteins forms intricate networks, including microtubules, which are fundamental for cellular structure, function, and genome stability [[2], [3], [4]]. Among tubulins, γ-tubulin (TUBG) plays a critical role in regulating microtubule formation by nucleating αβ-tubulin dimers [[5], [6], [7]] within the γ-tubulin ring complex (γ-TuRC), a highly conserved multi-protein assembly that serves as a scaffold for microtubule nucleation [8]. Beyond microtubule nucleation, recent studies have highlighted the broad impact of the TUBG meshwork on various cellular processes, including cytoskeletal organization, energy production [9,10], and genomic integrity maintenance [3,11].

TUBG is abundantly found in centrosomes [5], where it not only nucleates microtubules [7] but also organizes actin [12] and vimentin [13] networks. TUBG interacts with the Arp2/3 complex, a key regulator of actin filament nucleation, and its activator WASH. These interactions facilitate TUBG's role in actin remodeling, particularly at actin-based membrane protrusions where the Arp2/3 complex operates. Notably, overexpression of TUBG inhibits the formation of actin stress fibers without affecting microtubule organization, suggesting that TUBG has a distinct regulatory role in actin dynamics [14]. Additionally, TUBG associates with chromatin, contributing to the assembly of laminB at the nuclear envelope [3]. This nucleation function is essential for the formation of the mitotic spindle during cell division [5]. Cells deficient in TUBG do not survive, underscoring its indispensable role in cellular viability [9].

In previous work, we identified a nuclear localization signal and DNA-binding domain within the C-terminal region (TUBG^334-451^) of TUBG, as well as a GTPase domain in the N-terminal region (TUBG^1-335^) [3,4,9]. The N-terminal GTPase domain is critical for mitochondrial dynamics and spatial organization, while the C-terminal DNA-binding domain is involved in chromatin interactions and lamin B association [3,4,9]. Together, these regions underscore TUBG's multifunctionality in maintaining cellular architecture, coordinating nuclear integrity, and organizing various cytoskeletal components [3,7,12,13,15]. This hubbing property may be crucial for preserving cellular homeostasis and responding to structural perturbations.

This study investigates the molecular influence of the TUBG meshwork on cytoskeletal integrity with a particular focus on the functional contributions of its N- and C-terminal regions. Our findings indicate that reduced TUBG levels lead to alterations in the integrity of microtubules, vimentin, and laminB, while also reinforcing the actin network. Moreover, expressing either the N- or the C-terminal regions of TUBG in cells with reduced TUBG levels led to distinct effects on the stability of microtubules, vimentin, actin, and laminB. Understanding the molecular interactions between TUBG1 and these cellular components is crucial for elucidating its role in both health and disease [16].

## 2 Materials and methods

### 2.1 Chemicals and reagents

The following antibodies and reagents were utilized in the study: anti-TUBG (1:900 rabbit polyclonal, cat. No. T3320 and T5192; mouse monoclonal, cat. No. T6557) from Sigma-Aldrich, Alexa Fluor™ 568 Phalloidin (1:150; Cat no: A12380) from Thermo Fisher Scientific; and anti-α-tubulin (1:1500 mouse monoclonal; cat. No. CP06) from Calbiochem, anti-vimentin (1:1800 mouse monoclonal; cat. No. sc-373717) and anti-laminB (1:1800 goat polyclonal; cat. No. sc-6216) both from Santa Cruz Biotechnology.

The anti-TUBG antibodies recognized the following TUBG sequences: T3320, raised against amino acids at the C-terminus of *Xenopus* TUBG, and T5192 and T6557, raised against amino acids 38–53 at the N-terminus of TUBG.

Human *TUBG1* single guide (sgRNA; Addgene plasmid 104437), TUBG_rest_^1-335^ (*TUBG1*sgRNA resistant; Addgene plasmid 226519) and TUBG^334-451^ (Addgene plasmid 104436) were prepared as previously reported [17].

### 2.2 Cell culture

U2OS cells (cat. No. HTB-96 from ATCC) and A549 (cat. No. CCL-185 from ATCC) were cultured and transfected with X-fect (Takara Bio Europa, Cat. No. 631317), according to the manufacturer's instructions [18], using *TUBG1* sgRNA or co-expressing *TUBG1* sgRNA and either *TUBG*_rest_^1-335^- or *TUBG*^334-451^- sg-resistant genes (1:1). One day after transfection, 40,000 cells were plated on 35 mm glass-bottom dishes (MatTek, Cat. No. p35G-1.0-20-C) and incubated for 7 days (*TUBG1* sgRNA) or 11 days (*TUBG1* sgRNA/TUBG_rest_^1-335^; sgRNA/TUBG^334-451^) before fixation. The culture medium was changed every second day during the incubation period. To minimize cell death, we avoided passaging Cas9-GFP-*TUBG1-*sg-expressing cells, as they are particularly sensitive to excessive handling. *TUBG1* sgRNA-expressing cells were fixed after 7 days, as this time point was previously shown to minimize cell loss due to death while achieving the lowest TUBG expression levels [9].

### 2.3 Microscopy

U2OS cells were cultured and fixed as described previously [11,19]. The fixation procedure involved a two-step process: first using paraformaldehyde, followed by a final fixation and permeabilization step using a mixture of methanol and acetone (1:1) [19]. Confocal fluorescence imaging studies were conducted using a Zeiss LSM 700 Axio Observer microscope equipped with a Plan-Apochromat × 40 or × 63 NA 1.40 oil immersion objective. For all images presented in this paper, a rolling ball background subtraction was applied using Fiji. Image analysis, Z-stack projections, and further image processing were performed using the ImageJ2 (Fiji, version 2.14.0/1.54f) software [20].

The fluorescence intensity for actin, α-tubulin, and vimentin was measured across the entire cell area, while for lamin B, only the intensity within the nuclear compartment was analyzed. These measurements were performed using maximum-intensity projections of sequential images, with regions of interest manually defined based on the respective staining patterns. The fluorescence intensity values presented in the manuscript correspond to the raw data obtained directly from ImageJ software. All measurements were conducted in a consistent manner across samples to ensure accuracy and reproducibility. The thickness of the sections used for Z-stack projections was 0.4 μm.

### 2.4 Statistical analysis

Data are presented as means ± standard derivation (SD), and the statistical comparisons were performed to assess differences between groups. The normality of the data was evaluated using the Shapiro-Wilk test. The results indicated that most data sets did not meet the normality assumption required for the *t*-test (e.g., p-values below 0.05 in X out of Y cases). Based on these findings, the Wilcoxon matched-pairs signed-rank test was used for paired samples, and the Mann-Whitney *U* test was used for unpaired samples. Statistical significance was determined as follows: ∗*P* < 0.05, ∗∗*P* < 0.01, ∗∗∗*P* < 0.0005, ∗∗∗∗*P* < 0.0001.

## 3 Results

### 3.1 Reducing cellular expression of TUBG

The RNA-single guide (sg)-mediated reduction of TUBG1 expression posed experimental challenges, often requiring over a week to achieve a decrease below 50 % [18,21]. This reduction has been shown to impair cell proliferation rates [18,21], which can be restored by the expression of a sg-resistant *TUBG1* gene [17].

**Fig. 1:**
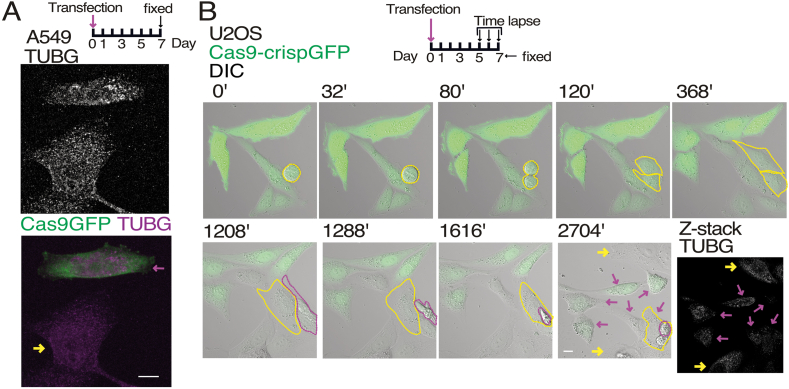
*TUBG1* knockdown induces cytotoxicity. (A) Confocal images of A549 cells expressing *TUBG1*-sg (Cas9-crispGFP; green). Cells were transfected on day 0, fixed after seven days, and immunostained with an anti-TUBG antibodies. A schematic representation of the experimental timeline is included for context. Control cells (non-expressing Cas9-crispGFP) are marked with yellow arrows, and *TUBG1*-sg-expressing cells with magenta arrows. Whole-cell confocal images were captured, with Z-stacks processed into maximum-intensity projections (*n* = 4 experiments). Scale bars: 10 μm. (B) Time-lapse and confocal imaging of U2OS cells expressing *TUBG1*-sg (Cas9-crispGFP; green). A schematic representation of the time-lapse experiments is provided. Time-lapse imaging began five days post-transfection, with cell populations monitored for two days prior to fixation. Representative merged differential interference contrast (DIC) and fluorescence images from time-lapse sequences are shown. Images were captured every 8 min. In the displayed series, seven cells are visible; one is undergoing division (outlined with yellow dashed lines), and one of its offspring subsequently undergoing cell death (outlined with magenta dashed lines; *n* = 4 experiments). Cells were fixed after seven days, and immunostained with anti-TUBG antibodies. Control cells (non-expressing Cas9-crispGFP) are marked with yellow arrows, and *TUBG1*-sg-expressing cells with magenta arrows. Whole-cell confocal images were captured, with Z-stacks processed into maximum-intensity projections. Scale bars: 10 μm.

While cells expressing *TUBG*-short hairpin (sh) could establish a stable cell line with a proximately 50 % reduction in TUBG expression [18,21], this differs for cells expressing *TUBG1*-sg, as Cas9-sg-mediated knockout totally impeded the expression of TUBG1 [22]. We gene-edited various cell lines, including A549 (adenocarcinomic alveolar basal epithelia) and U2OS (human osteosarcoma) cells, using a green fluorescence tagged Cas9- *TUBG1*-sg gene (Fig. 1). In most cell lines, we were unable to significantly reduce the endogenous expression of TUBG1 (Fig. 1A), with the exception of U2OS cells (Fig. 1B). After seven days, several outcomes were observed in Cas9- *TUBG1*-sg-expressing U2OS cells: 1. Non-transfected cells outgrew GFP-crispCas9-positive cells, dominating the population. 2. Many cells underwent apoptosis due to decreased TUBG levels (Fig. 1B). 3. Despite carrying the Cas9-GFP-*TUBG-*sg plasmid, some cells evaded Cas9 action and maintained 100 % TUBG levels. 4. Following *TUBG1-*sg transfection, cells initially divided but subsequently remained in interphase before dying (Fig. 1B), confirming that TUBG depletion is cytotoxic [9,23].

### 3.2 Decreased TUBG expression affects microtubule formation

**Fig. 2:**
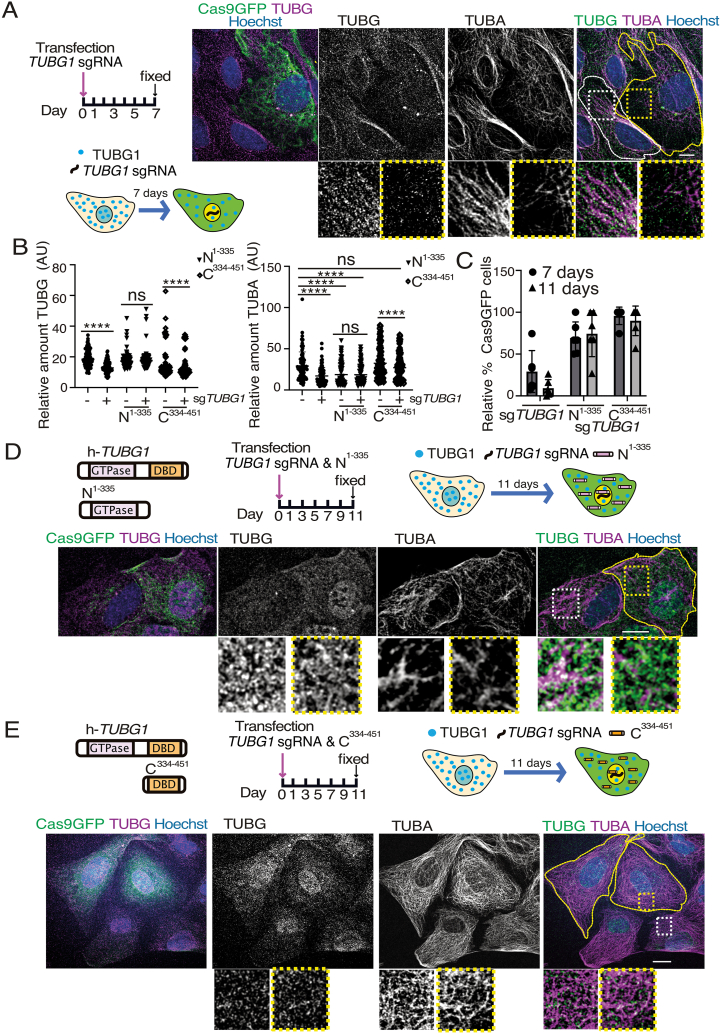
Microtubule disruption in *TUBG1*-sg expressing cells. (A–E) Overview and Experimental Design: U2OS cells were analyzed under different conditions to assess microtubule disruption. A schematic representation of the experimental timeline is included for context (A, D & E). Fixed cells were immunostained with anti-TUBG and anti-α-tubulin (TUBA) antibodies, with DNA counterstained using Hoechst. Confocal microscopy was used to acquire Z-stacks, and maximum-intensity projections were generated for visualization. Cells expressing Cas9-GFP-*TUBG1*-sgRNA (Cas9GFP) are outlined by yellow dashed lines. Magnified insets show representative regions of control cells (white boxes) and Cas9-GFP-*TUBG1*-sgRNA expressing cells (yellow boxes). Scale bars: 10 μm. (A) Experimental Condition 1: Cells expressing Cas9-GFP-*TUBG1*-sgRNA (*n* = 90) were compared to Cas9-GFP-*TUBG1*-sg-non-expressing control cells (*n* = 90; *n* = 8 experiments). (B) The graphs show the fluorescence intensity of TUBG and TUBA measured using ImageJ. Statistical significance is indicated as ns = non-significant; ∗∗∗∗P < 0.0001 (paired/unpaired Wilcoxon matched/Mann-Whitney test). (C) The graph shows the relative percentage of cells expressing Cas9-GFP over time. The sample with the largest number of Cas9-GFP-expressing cells was set to 100 %, and values at other time points were normalized (*n* = 6 experiments). (D, E) Experimental Condition 2: Cells expressing *TUBG1*-sgRNA-resistant constructs, *TUBG*^1-335^ (D; N^1-335^) or *TUBG*^334-451^ (E; C^334-451^), were used as additional controls. These were compared to cells co-expressing both Cas9-GFP-*TUBG1*-sgRNA and the respective resistant constructs. Fluorescence intensity in control cells expressing only *TUBG*^1-335^ (*n* = 102) or *TUBG*^334-451^ (*n* = 94) was compared to cells co-expressing Cas9-GFP-*TUBG1*-sgRNA with the respective resistant constructs (*n* = 102 and *n* = 94, respectively; *n* = 3 experiments).

Microtubule nucleation is initiated by TUBG, a key component of the γTURC, which is localized at microtubule-organizing centers like the centrosomes. The γTURC serves as a scaffold, stabilizing the minus end of microtubules and facilitating the addition of αβ-tubulin dimers. This process is essential for proper microtubule formation and organization, which are critical for cellular functions such as cell division [24]. To investigate the effect of impaired TUBG expression on microtubules, we expressed Cas9-GFP-*TUBG1-*sg in U2OS cells for seven days and used immunofluorescence staining with antibodies against α-tubulin (a microtubule marker) and TUBG (Fig. 2A and B). Cells with reduced TUBG expression exhibited disrupted microtubule networks, with a decreased number of microtubules concentrated around the nuclear region (Fig. 2A and B). Quantitative analysis revealed that the mean intensity of α-tubulin was reduced by 38.52 % in Cas9-GFP-*TUBG1-*sg-expressing cells compared to non-expressing cells (Fig. 2B & Table 1). Our findings highlight the critical role of TUBG in maintaining microtubule integrity.

**Table 1:** The table presents the average percentage (±SD) of fluorescence intensity measured via immunofluorescence staining of the indicated network protein. Fluorescence intensities were normalized to control U2OS cells (non-expressing Cas9-GFP-*TUBG1*-sgRNA) or to control cells expressing either *TUBG*^1-335^ or *TUBG*^334-451^, with control values set to 100 %. The data include average fluorescence intensities from cells expressing Cas9-GFP-*TUBG1*-sgRNA, as well as from cells co-expressing Cas9-GFP-*TUBG1*-sgRNA with either *TUBG*^1-335^ or *TUBG*^334-451^.

Cellular Structural Network	Average Fluorescence Intensity (%)		
	Cas9-GFP-*TUBG1*-sgRNA	*TUBG*^1-335^ Cas9-GFP-*TUBG1*-sgRNA	*TUBG*^334-451^ Cas9-GFP-*TUBG1*-sgRNA
Microtubules	61.48 ± 22.48	109.76 ± 37.38	87.24 ± 22.40
Vimentin	65.13 ± 12.16	114.80 ± 44.45	111.96 ± 21.00
Lamin B	85.24 ± 25.43	137.43 ± 70.03	116.36 ± 26.28
Actin	131.29 ± 48.68	99.58 ± 23.78	113.84 ± 22.78

### 3.3 Microtubule formation is reestablished by the expression of C-terminal TUBG^334-451^

Given the critical roles of the N- and C-terminal regions of TUBG1 in cellular function, we investigated whether expressing these regions could compensate for TUBG1 depletion in U2OS cells. Although the expression of full-length sg-resistant TUBG1 has been shown to rescue microtubule assembly and cellular survival [17], neither the N-terminal (TUBG^1-335^) nor the C-terminal (TUBG^334-451^) fragments fully reversed the lethal effects induced by Cas9-GFP-*TUBG1-*sg [17]. However, both fragments extended the survival of TUBG1 depleted cells, suggesting that they could temporarily mitigate the detrimental effects of TUBG1 depletion (Fig. 2C). To evaluate their impact on microtubule network integrity, we co-expressed the N- or C-terminal fragments with Cas9-GFP-*TUBG1* for 11 days, a time point at which TUBG1 depletion severely disrupts cellular structure (Fig. 2C). Expression of TUBG^1-335^ resulted in reduced microtubule intensity, regardless of Cas9-GFP-*TUBG1-*sg co-expression, indicating that TUBG^1-335^ disrupts microtubule assembly (Fig. 2 B&D). Although microtubule intensity was lower compared to non-expressing cells (Fig. 2 A), cells expressing TUBG^1-335^ displayed a more even distribution of microtubules than those expressing Cas9-GFP-*TUBG1-*sg (Fig. 2A and D and Table 1).

In contrast, the expression of TUBG^334-451^ successfully restored microtubule formation in cells expressing Cas9-GFP-*TUBG1-*sg (Fig. 2B and E). The effect of TUBG^334-451^ on microtubule restoration was even more pronounced in non-expressing Cas9-GFP-*TUBG1-*sg cells (Fig. 2 B&E and Table 1). Altogether, these findings demonstrate that the C-terminal region of TUBG1 is necessary for microtubule assembly.

### 3.4 Vimentin organization is affected by a decreased TUBG expression

**Fig. 3:**
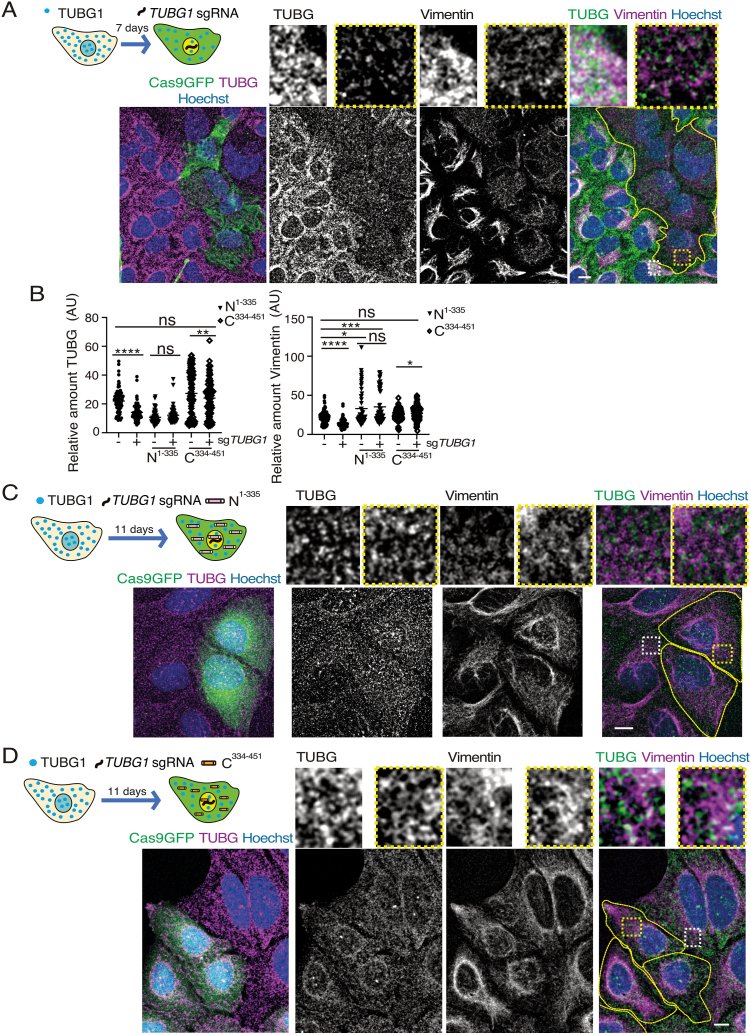
Impact of *TUBG1*-sg expression on the vimentin network. (A–D) Overview and Experimental Design: Confocal microscopy was used to assess the effects of *TUBG1*-sgRNA expression on the vimentin network in U2OS cells. A schematic representation of the experimental timeline is included for context (A, C &D). Fixed cells were immunostained with anti-TUBG and anti-vimentin antibodies, and DNA was counterstained using Hoechst. Maximum-intensity projections of Z-stacks were generated to visualize the entire cell structure. Cells expressing Cas9-GFP-*TUBG1*-sgRNA are outlined by yellow dashed lines. Magnified insets show representative regions of control cells (white boxes) and *TUBG1*-sgRNA expressing cells (yellow boxes). Scale bars: 10 μm. (A) Experimental Condition 1: Cells expressing Cas9-GFP-*TUBG1*-sgRNA (Cas9GFP; *n* = 100) were compared to control cells (non-expressing Cas9-GFP; *n =*100; *n* = 4 experiments). (B) Quantification: Fluorescence intensity of TUBG and vimentin was measured using ImageJ. Statistical significance is indicated as ns = non-significant; ∗P < 0.05, ∗∗P < 0.01, ∗∗∗P < 0.0005, ∗∗∗∗P < 0.0001 (paired/unpaired Wilcoxon matched/Mann-Whitney test). (C, D) Experimental Condition 2: Cells co-expressing *TUBG1*-sgRNA-resistant constructs were analyzed: TUBG^1-335^ (C) or *TUBG*^334-451^ (D) alongside *TUBG1*-sgRNA. In panel (C), fluorescence intensity was compared between cells expressing *TUBG*^1-335^ alone (*n* = 55) and cells co-expressing *TUBG*^1-335^ and *TUBG1*-sgRNA (*n* = 55; *n* = 4 experiments). In panel (D), fluorescence intensity was compared between cells expressing *TUBG*^334-451^ alone (*n* = 102) and cells co-expressing *TUBG*^334-451^ and *TUBG1*-sgRNA (*n* = 102; *n* = 4 experiments).

In addition to microtubules, cells contain intermediate filaments such as vimentin, which primarily provide mechanical strength to cells and tissues [13]. Vimentin fibers are known to nucleate at centrosomes [13], and interactions between TUBG and vimentin has been reported [17]. To build on these findings, we investigated the potential consequences of impaired TUBG expression on vimentin network formation. To explore this, we induced Cas9-GFP-*TUBG1-*sg expression in U2OS cells and assessed its impact on vimentin organization by immunofluorescence staining of both vimentin and TUBG after seven days of Cas9-GFP-*TUBG1-*sg expression (Fig. 3A). Cells with reduced TUBG expression displayed a disrupted vimentin network, characterized by fewer vimentin fibers in the cytoplasm (Fig. 3A), suggesting that TUBG plays a critical role in maintaining vimentin filament organization.

### 3.5 Vimentin network formation is reestablished by the expression of either TUBG^1-335^ or TUBG^334-451^

While microtubules exhibit dynamic instability, growing and shrinking rapidly, vimentin filaments differ from microtubules in their assembly process. Vimentin are made of monomers that laterally associate and end-to-end anneal to form a mature, non-polar structure, in contrast to actin and microtubules where monomer addition occurs primarily at the filament's ends [25].

To investigate how TUBG contributes to the maintenance of the vimentin network, we co-expressed Cas9-GFP-*TUBG1-*sg with either TUBG^1-335^ or TUBG^334-451^ fragments for 11 days (Fig. 3B–D). Expression of either the N-terminal (TUBG^1-335^) or the C-terminal (TUBG^334-451^) regions of TUBG1 reversed the effects on the vimentin network caused upon reduced TUBG-levels (Fig. 3A–D). Notably, the expression of TUBG^1-335^ increased vimentin intensity, above the levels observed in non-expressing Cas9-GFP-*TUBG1-*sg control U2OS cells (Fig. 3A and B&C and Table 1), indicating that the TUBG^1-335^ fragment has a stronger effect in stabilizing vimentin filaments. Nonetheless, the recovered meshwork does not resemble the wild-type network 100 %, as the missing fragments are most likely involved in the fine-tuning of the meshwork organization. The data confirm a functional relationship between the TUBG and the vimentin network, emphasizing the importance of TUBG in maintaining cytoskeletal integrity [13].

### 3.6 Diminished TUBG expression impairs the nuclear envelope

**Fig. 4:**
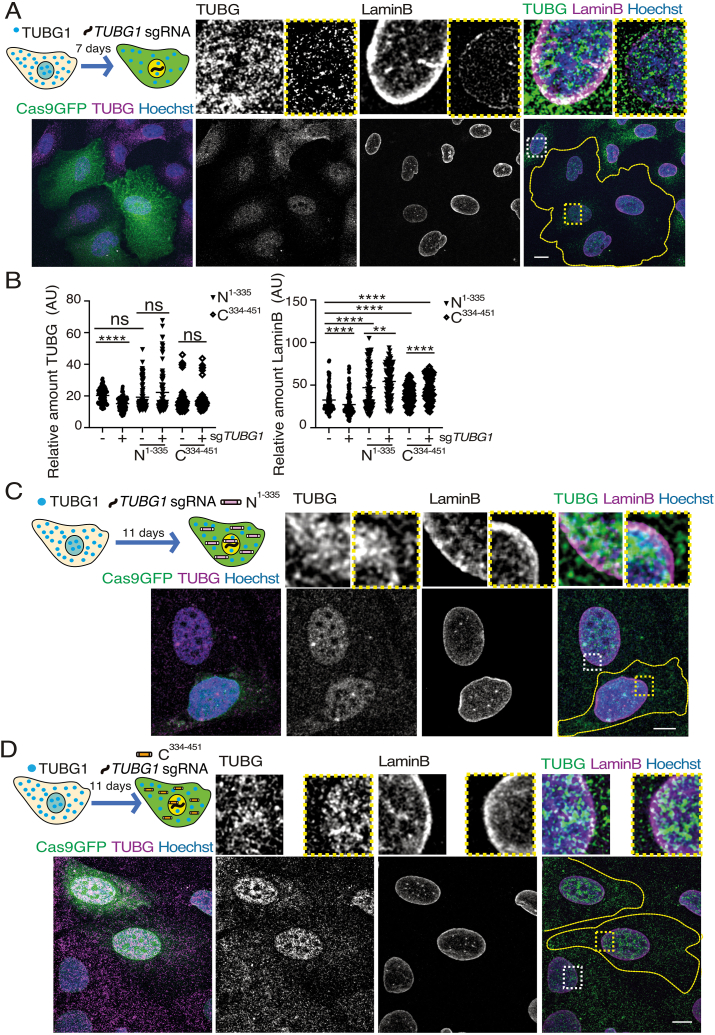
Altered nuclear envelope in *TUBG1*-sg-expressing cells. (A–D) Overview and Experimental Design: Confocal microscopy was used to assess the effects of *TUBG1*-sgRNA (Cas9GFP) expression on the nuclear envelope in U2OS cells. A schematic representation of the experimental timeline is included for context (A, C &D). Fixed cells were immunostained with anti-TUBG and anti-laminB antibodies, and DNA was counterstained using Hoechst. Z-stacks were processed into maximum-intensity projections for analysis. Cells expressing Cas9-GFP-*TUBG1*-sgRNA are outlined by yellow dashed lines. Magnified insets highlight representative regions of control cells (white boxes) and *TUBG1*-sgRNA expressing cells (yellow boxes). Scale bars: 10 μm. (A) Experimental Condition 1: Cells expressing Cas9-GFP-*TUBG1*-sgRNA (*n* = 116) were compared to control cells (non-expressing Cas9-GFP; *n* = 116; *n* = 8 experiments). (B) Quantification: Fluorescence intensity of TUBG and laminB was measured using ImageJ. Statistical significance is indicated as ns = non-significant; ∗∗P < 0.01, ∗∗∗∗P < 0.0001 (paired/unpaired Wilcoxon matched/Mann-Whitney test). (C, D) Experimental Condition 2: Cells co-expressing *TUBG1*-sgRNA-resistant constructs were analyzed: *TUBG*^1-335^ (C) or *TUBG*^334-451^ (D), alongside *TUBG1*-sgRNA expression. In panel (C), fluorescence intensity was compared between cells expressing *TUBG*^1-335^ alone (*n* = 100) and cells co-expressing *TUBG*^1-335^ and *TUBG1*-sgRNA (*n* = 100; *n* = 3 experiments).In panel (D), fluorescence intensity was compared between cells expressing *TUBG*^334-451^ alone (*n* = 103) and cells co-expressing *TUBG*^334-451^ and *TUBG1*-sgRNA (*n* = 103; *n* = 3 experiments).

Previous studies conducted by our research group have shed light on TUBG's pivotal role in chromatin organization and nuclear assembly, demonstrating its involvement in recruiting the intermediate filament protein lamin B and facilitating nuclear membrane formation [3]. To investigate the impact of an impaired TUBG expression on the nuclear envelope, we induced Cas9-GFP-*TUBG1-*sg expression in U2OS cells during seven days and evaluated nuclear envelope integrity using immunofluorescence staining for lamin B, a marker for nuclear lamina (Fig. 4A and B). Our analysis revealed that cells with decreased TUBG expression exhibited impaired lamin B recruitment to the nuclear envelope (Fig. 4A and B), underscoring the critical role of TUBG in preserving nuclear integrity and emphasizing additional cellular damages associated with reduced TUBG levels.

### 3.7 Lamin B intensity is reestablished by the expression of either TUBG^1-335^ or TUBG^334-451^

Lamin B is a key component of the nuclear lamina, crucial for maintaining nuclear structure and regulating chromatin organization. Gene duplications of *lamin B1* can cause autosomal dominant leukodystrophy, a neurodegenerative disease characterized by demyelination [26]. In cancers, lamin B1 expression are associated with promoting tumor aggressiveness. Lamin B1 is also linked to cellular senescence and premature aging, where its reduction disrupts nuclear architecture and accelerates aging [27].

To explore how TUBG supports the maintenance of lamin B, we co-expressed Cas9-GFP-*TUBG1-*sg with either TUBG^1-335^ or TUBG^334-451^ fragments for 11 days (Fig. 4). Both TUBG1 fragments restored the reduced lamin B levels caused by disminished TUBG expression, exceeding the levels observed in non-expressing Cas9-GFP-*TUBG1-*sg control U2OS cells (Fig. 4A–D and Table 1). This data highlights a functional relationship between the TUBG and lamin B, underscoring the critical role of TUBG in maintaining nuclear integrity [3].

### 3.8 Altered actin dynamic upon diminished TUBG expression

**Fig. 5:**
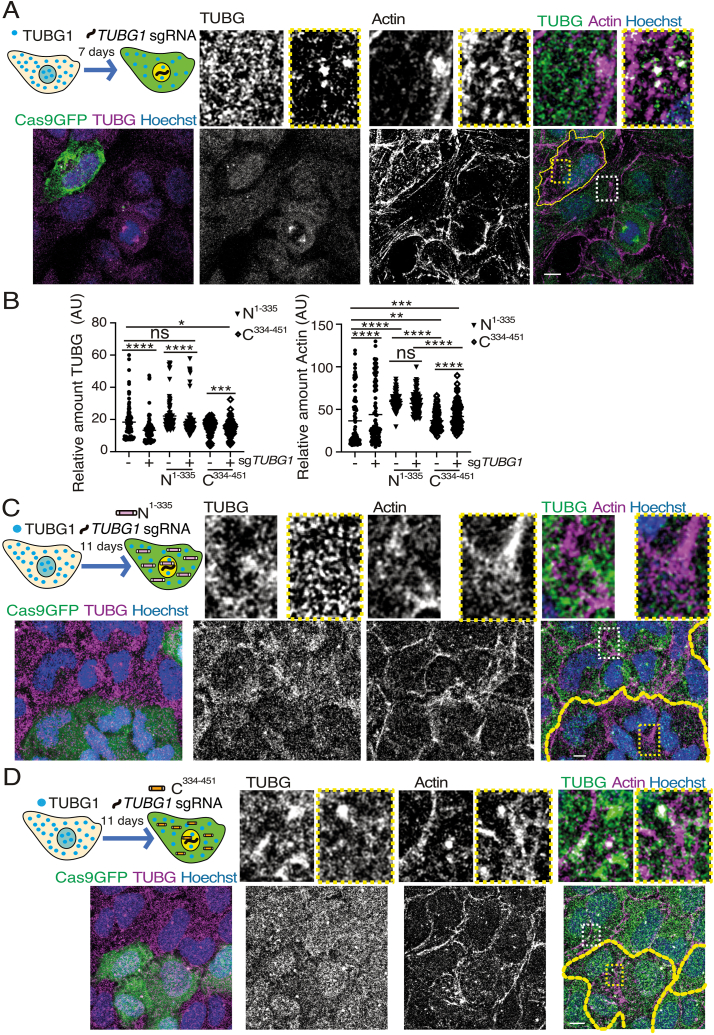
Effects of *TUBG1*-sg expression on actin organization. (A–D) Overview: Confocal microscopy was used to examine the impact of *TUBG1*-sgRNA (Cas9GFP) expression on the actin cytoskeleton in U2OS cells. A schematic representation of the experimental timeline is included for context (A, C &D). Cells were immunostained with anti-TUBG antibodies and phalloidin to visualize actin, with DNA counterstained using Hoechst. Z-stacks were processed into maximum-intensity projections to capture the entire cell structure. Cells expressing Cas9-GFP-*TUBG1*-sgRNA are outlined by yellow dashed lines. Insets highlight magnified regions (white boxes for controls, yellow boxes for *TUBG1*-sgRNA-expressing cells). Scale bars: 10 μm. (A) Experimental Condition 1: Cells expressing Cas9-GFP-*TUBG1*-sgRNA (*n* = 100) were compared to control cells (non-expressing Cas9-GFP; *n* = 100; *n* = 4 experiments). (B) Quantification: Fluorescence intensity of TUBG and actin was quantified using Image J. Statistical significance was assessed as follows: ns = non-significant; ∗P < 0.05, ∗∗P < 0.01, ∗∗∗P < 0.0005, ∗∗∗∗P < 0.0001 (paired/unpaired Wilcoxon matched/Mann-Whitney test). (C) Experimental Condition 2: Cells co-expressing *TUBG1*-sgRNA and the *TUBG1*-sgRNA-resistant construct *TUBG*^1-335^ were analyzed. Fluorescence intensity was compared between cells expressing *TUBG*^1-335^ alone (*n* = 55) and cells co-expressing *TUBG*^1-335^ and TUBG1-sgRNA (*n* = 55; *n* = 3 experiments). (D) Experimental Condition 3: Cells co-expressing *TUBG1*-sgRNA and the *TUBG1*-sgRNA-resistant construct *TUBG*^334-451^ were analyzed. Fluorescence intensity was compared between cells expressing *TUBG*^334-451^ alone (*n* = 102) and cells co-expressing *TUBG*^334-451^ and TUBG1-sgRNA (*n* = 102; *n* = 3 experiments).

Actin has been observed to associate with the TUBG-ring complex, determining its geometry [28]. Additionally, the TUBG-rich centrosome serves as a nucleating site for actin fibers [12]. Moreover, both actin and TUBG are localized in the nucleus and mitochondria [29,30], suggesting a potential interdependence between these cytoskeletal components. To explore how an impaired TUBG expression affects actin filament organization at specific time points, we induced Cas9-GFP-*TUBG1-*sg expression in U2OS cells and used phalloidin staining to visualize actin, alongside TUBG immunofluorescence, seven days post-transfection (Fig. 5A and B). Cells with a reduced TUBG expression showed alterations in the actin network, exhibiting increased intensity, which reflects a reinforced actin network (Fig. 5A and B). These findings confirm a connection between TUBG and the actin network, highlighting the interplay between these cytoskeletal systems.

### 3.9 The C-terminal region of TUBG connects the networks

Given the many shared biological functions between actin and TUBG within the cell [12,[28], [29], [30]] and the observed reinforcement of the actin network upon reduced TUBG levels, it is crucial to identify which region of the TUBG protein that mediates the compensatory effect of actin in the absence of TUBG. Understanding this interaction could provide valuable insights into the functional interdependence between these cytoskeletal components.

Co-expression of human *TUBG1* sgRNA with either TUBG_rest_^1-335^ or TUBG^334-451^ fragments revealed that TUBG_rest_^1-335^ expression increased actin network intensity, even in the absence of Cas9-GFP-*TUBG1-*sg co-expression. This finding indicates that the N-terminal of TUBG1 promotes an increase in actin filaments (Fig. 5B and C). Similarly, expression of the C-terminal fragment (TUBG^334-451^) also enhanced actin network intensity, but this increase was significantly lower (∗∗∗*P* < 0.0001) compared to cells expressing TUBG_rest_^1-335^ (Fig. 5A–D and Table 1). Collectively, these results identify the C- and N-terminal regions of TUBG1 as critical domains that mediate the connection between the actin and TUBG meshworks [12,28].

## 4 Discussion

The organization of cellular components into distinct compartments is crucial for maintaining cellular functions and homeostasis [15]. In this study, we aimed to elucidate the molecular mechanisms by which TUBG1 coordinates cytoskeletal networks and cellular organization.

Our results demonstrate that reducing TUBG1 expression posed significant experimental challenges, including impaired cell proliferation and increased cytotoxicity, consistent with previous studies [9,31]. This highlights the essential role of TUBG1 in cell survival and homeostasis. Specifically, TUBG1 depletion disrupted microtubule formation, leading to severe alterations in microtubules and resulting in growth arrest [32]. These findings emphasize TUBG1's critical function in maintaining microtubule integrity, a vital aspect of cellular health [31].

In addition to microtubules, reduced TUBG1 expression also impacts the organization of other cytoskeletal elements, such as vimentin, and disrupts the structural integrity of the nuclear envelope. The altered vimentin organization confirms TUBG1's role in intermediate filament integrity. The observed defects in the nuclear envelope, with reduced lamin B recruitment, further underscore TUBG1's involvement in nuclear integrity and chromatin organization [3,11]. These changes may be linked to G1 phase arrest, which occurs upon TUBG depletion [11,21].

In parallel with these changes, we observed significant alterations in actin organization following TUBG1 depletion, with actin filaments becoming more pronounced. This likely represents a compensatory response to the loss of TUBG1. The observed compensatory increase in actin filament intensity could be an attempt by the cell to restore cellular architecture and counterbalance the broader cytoskeletal disruptions caused by TUBG loss. Interestingly, this finding aligns with Hubert and coworkers' observation that TUBG overexpression inhibits actin stress fiber formation [14]. The contrasting effects of TUBG1 depletion, which reinforce actin filaments, and TUBG overexpression, which suppresses them, highlight a regulatory role for TUBG1 in actin filament organization. These findings suggest that under normal conditions, TUBG1 suppresses actin filament assembly, while its loss triggers a compensatory increase in actin filament intensity. This increase in actin polymerization could serve as a cellular adaptation to maintain structural integrity in the face of microtubule instability and other cytoskeletal perturbations. The broader disruptions across multiple cytoskeletal components—including microtubules, actin filaments, and intermediate filaments—further underscore TUBG1's role as an integrative hub for cytoskeletal regulation.

The C-terminal TUBG^334-451^ fragment was crucial for restoring microtubule integrity, while both N- and C-terminal fragments compensated for TUBG1 loss in intermediate filament networks, such as vimentin and lamin B. This suggests that intermediate filaments may rely on a more structural role of TUBG1, allowing both fragments to compensate. In contrast, in dynamic networks like microtubules, the C-terminal region is essential for maintaining integrity, while the N-terminal, containing the GTPase domain, likely plays a regulatory role in coordinating TUBG1's interaction with microtubules [12,[28], [29], [30]].

One puzzling aspect of our findings is that the N-terminal fragment of TUBG1, despite containing the essential GTPase domain [17], failed to restore microtubule assembly or rescue cell viability in the absence of full-length TUBG1. While the GTPase activity in the N-terminal region is suggested to be involved in microtubule nucleation [4], our results suggest that it is insufficient on its own to compensate for the loss of TUBG1. This insufficiency likely arises from the additional structural or regulatory roles provided by the C-terminal region. The C-terminal region harbors domains responsible for nuclear localization, DNA binding, and interactions with other cytoskeletal elements, offering scaffolding and regulatory functions that complement the enzymatic activity of the GTPase domain. The coordinated contribution of both regions appears essential for stabilizing and organizing the microtubule network within the cellular context.

Furthermore, the C-terminal region's interactions with other cellular components, such as actin and intermediate filaments, may be important for integrating the microtubule network with broader cytoskeletal systems. The absence of these interactions in the N-terminal fragment underscores the hubbing nature of TUBG's regulatory and structural roles across multiple cytoskeletal networks.

It is important to note, however, that our study primarily quantified the overall content of respective proteins across the cell. While this approach provides valuable insights into the effects of TUBG1 depletion on cytoskeletal elements, it does not address changes in the spatial distribution or morphological features of these proteins. Furthermore, the study does not specifically analyze how the different filamentous elements of the TUBG meshwork—namely γ-tubules, γ-strings, and centrosomes [3,17,33]—contribute to the observed effects on other cytoskeletal components. Understanding the distinct roles and interactions of these individual TUBG structures could provide critical insights into their specific contributions to cytoskeletal organization and dynamics. Future studies focused on these aspects could further refine our understanding of the interplay between TUBG1 and cytoskeletal components. This limitation highlights the need to complement biochemical and molecular analyses with spatial and morphological evaluations to capture the full spectrum of TUBG1's regulatory roles.

In conclusion, our findings provide important insights into the multifunctional role of TUBG1 in coordinating cytoskeletal elements, maintaining nuclear integrity, and preserving genomic stability. The inability of the N-terminal fragment to fully restore microtubule function suggests that while TUBG1's GTPase activity is necessary, it is not sufficient for its broader role in cytoskeletal coordination. Furthermore, the compensatory increase in actin filament organization observed upon TUBG1 depletion appears to reflect a response to the broader cytoskeletal and nuclear envelope disruptions caused by TUBG loss. Given these compensatory mechanisms, TUBG1-targeting strategies may hold therapeutic potential for disorders involving cytoskeletal dysfunction, such as certain types of cancer, neurodegeneration, and aging. Further research into these pathways could deepen our understanding of TUBG1's function in health and disease, potentially leading to innovative therapeutic strategies targeting TUBG-related pathways.

### Ethics declaration

This study was conducted using U2OS and A549 cell lines, which are widely established and commercially available. No direct experimentation on living beings was performed. The use of these cell lines complies with institutional and international ethical guidelines, and no additional ethical committee approval was required.

## CRediT authorship contribution statement

**Darina Malycheva:** Writing – review & editing, Writing – original draft, Validation, Methodology, Formal analysis, Conceptualization. **Maria Alvarado-Kristensson:** Writing – review & editing, Writing – original draft, Supervision, Resources, Project administration, Methodology, Investigation, Funding acquisition, Formal analysis, Data curation, Conceptualization.

## Data availability statement

Data associated with this study is include in the article, supplementary materials, or referenced within the text.

## Declaration of generative AI and AI-assisted technologies in the writing process

During the preparation of this work the authors used ChatGTP in order to improved the language. After using this tool, the authors reviewed and edited the content as needed and takes full responsibility for the content of the publication.

## Funding statement

This work was supported by the 10.13039/501100002794Swedish Cancer Society and the Skane University Hospital in Malmö Cancer Research Fund.

## Declaration of Competing Interest

The authors declare the following financial interests/personal relationships which may be considered as potential competing interests: Maria Alvarado Kristensson reports financial support and administrative support were provided by 10.13039/501100003252Lund University. Maria Alvarado Kristensson reports a relationship with Lund University that includes: employment. If there are other authors, they declare that they have no known competing financial interests or personal relationships that could have appeared to influence the work reported in this paper.
